# Electronic Health Record Implementations and Insufficient Training Endanger Nurses’ Well-being: Cross-sectional Survey Study

**DOI:** 10.2196/27096

**Published:** 2021-12-23

**Authors:** Tarja Heponiemi, Kia Gluschkoff, Tuulikki Vehko, Anu-Marja Kaihlanen, Kaija Saranto, Sari Nissinen, Janna Nadav, Sari Kujala

**Affiliations:** 1 Finnish Institute for Health and Welfare Helsinki Finland; 2 University of Eastern Finland Kuopio Finland; 3 Aalto University Espoo Finland

**Keywords:** electronic health records, implementation, information systems, training, stress, cognitive failures, time pressure, registered nurses

## Abstract

**Background:**

High expectations have been set for the implementations of health information systems (HIS) in health care. However, nurses have been dissatisfied after implementations of HIS. In particular, poorly functioning electronic health records (EHRs) have been found to induce stress and cognitive workload. Moreover, the need to learn new systems may require considerable effort from nurses. Thus, EHR implementations may have an effect on the well-being of nurses.

**Objective:**

This study aimed to examine the associations of EHR-to-EHR implementations and the sufficiency of related training with perceived stress related to information systems (SRIS), time pressure, and cognitive failures among registered nurses. Moreover, we examined the moderating effect of the employment sector (hospital, primary care, social services, and others) on these associations.

**Methods:**

This study was a cross-sectional survey study of 3610 registered Finnish nurses in 2020. EHR implementation was measured by assessing whether the work unit of each respondent had implemented or will implement a new EHR (1) within the last 6 months, (2) within the last 12 months, (3) in the next 12 months, and (4) at no point within the last 12 months or in the forthcoming 12 months. The associations were examined using analyses of covariance adjusted for age, gender, and employment sector.

**Results:**

The highest levels of SRIS (adjusted mean 4.07, SE 0.05) and time pressure (adjusted mean 4.55, SE 0.06) were observed among those who had experienced an EHR implementation within the last 6 months. The lowest levels of SRIS (adjusted mean 3.26, SE 0.04), time pressure (adjusted mean 4.41, SE 0.05), and cognitive failures (adjusted mean 1.84, SE 0.02) were observed among those who did not experience any completed or forthcoming implementations within 12 months. Nurses who perceived that they had received sufficient implementation-related training experienced less SRIS (*F*_1_=153.40, *P*<.001), time pressure (*F*_1_=80.95, *P*<.001), and cognitive failures (*F*_1_=34.96, *P*<.001) than those who had received insufficient training. Recent implementations and insufficient training were especially strongly associated with high levels of SRIS in hospitals.

**Conclusions:**

EHR implementations and insufficient training related to these implementations may endanger the well-being of nurses and even lead to errors. Thus, it is extremely important for organizations to offer comprehensive training before, during, and after implementations. Moreover, easy-to-use systems that allow transition periods, a re-engineering approach, and user involvement may be beneficial to nurses in the implementation process. Training and other improvements would be especially important in hospitals.

## Introduction

Registered nurses form the largest group using health information systems (HIS) in health care. Therefore, the successful implementation of new HIS highly depends on nurses. High expectations have been set for the implementation of HIS in health care, for example, regarding increased efficiency. Indeed, a previous study shows that the implementation of intensive care unit information systems decreased the time nurses spent on documentation by over 30% and increased the time spent on direct patient care [1]. However, the outcomes of implementations may not always meet the high expectations [2]. Failure to understand users’ needs and support workflow are some reasons why implementations may fail [3].

Registered nurses’ work is often stressful and cognitively burdensome, and difficulties with HIS may induce extra stress and time pressure. Nurses have been found to prefer electronic health records (EHRs) over paper charts and think that EHR usage enhances nursing work but increases demand on work time and decreases the quality of care [4]. Among nurses, difficult-to-use EHRs have been associated with high time pressure and distress [5], and cognitive workload [6,7], which in turn have been associated with cognitive failures as well as lower patient safety [8].

Even though registered nurses are competent users of EHRs [9], the new systems that are implemented may be difficult and complicated to use especially in the beginning, thus becoming additionally demanding. This may lead to information chaos [10], which has been shown to result in decision-making errors and increased mental workload [10,11]. A previous study showed substantially increased cognitive workload related to new EHR implementation for nurses [6]. Nurses have been found to be most dissatisfied approximately 9 months after implementation, whereas their perceptions appeared to be more balanced after 18 months [12]. In another study, nurses reported greater acceptance of the EHR 12 months after implementation than after 3 months [13].

Education and training are essential prerequisites for successful HIS implementation [14]. The intricacies of new systems and changing functionalities may require nurses to allocate time and effort if they wish to master the new systems. However, the demands and pressures of care may not always afford nurses time to learn the new systems, and it is possible that not enough training and time have been allocated to this process [15,16]. Therefore, it may be a burden for nurses being forced to learn how to use the new systems effectively and efficiently, especially if they are not offered sufficient implementation-related training. Indeed, it has been found that HIS implementation may increase nurses’ workload if they receive insufficient training before implementation [14]. Training has even been shown to have a positive impact on the perceived work environment as well [17].

Stress is an ambiguous concept with many definitions; for example, it has been defined as a relationship between a person and the environment that is appraised as important for an individual and exceeds coping resources [18]. Poorly functioning and constantly changing information systems may elicit this kind of stress appraisal, which can be designated as stress related to information systems (SRIS). For example, information systems have emerged as one of the highest stress-inducing factors among Finnish physicians alongside time pressure and patient-related stress [19-21]. Previous findings show that SRIS has increased in the 21st century among physicians [19,21], and the usability of EHRs has an effect on its levels [22]. However, SRIS is less studied among nurses and more information is needed. For example, nurses themselves have proposed that training could be one way to reduce nurses’ SRIS levels [23].

As mentioned previously, studies show that nurses are dissatisfied after HIS implementations and challenges with HIS are associated with stress, time pressure, and cognitive burden [5-7,12,13,24]. Previous implementation studies have often focused on implementations involving transitions from paper-based systems to EHR systems [6,12]. However, many developed countries have already reached or will soon reach a saturation point where almost all health care organizations use EHRs. For example, in Finland, the EHR coverage has reached a saturation point of 100%, and many different brands of systems are in use [25]. Thus, more information is needed, especially on the effects of transitioning from one EHR system to another and implementation-related training on the stress levels and well-being of nurses. Therefore, our interest was on implementation of new brands of EHR systems, that is, the changes experienced when transitioning from one EHR to another.

Finnish nurses use many different EHR system brands; for example, in public hospitals, the 7 most popular brands are used by approximately 92% of nurses, and in primary care, the 4 most popular brands are used by 94% of nurses [26]. The most commonly used EHRs among nurses in Finland are Lifecare, Uranus, Pegasos, Apotti (system brand: Epic), Effica Healthcare, Mediatri, Esko, DynamicHealth, and DomaCare [26]. For example, large-scale implementations of Apotti were in progress in different areas of Helsinki and the Uusimaa region from 2018 to 2020.

This study examined the associations between EHR implementations and the sufficiency of training related to implementations with perceived SRIS, time pressure, and cognitive failures among Finnish registered nurses. Moreover, previous studies show that the employment sector, such as whether a person is employed in a hospital or a primary care center, plays an important role in health professionals’ perceptions of EHRs and how they affect professionals’ stress levels and well-being [22,27,28]. Therefore, we additionally examined whether the employment sector would have an effect on these associations.

## Methods

### Sample

The data were collected during the spring of 2020 through an internet-based Webropol survey. The link to the survey was sent via email by the Finnish Nurses Association, Tehy (The union of health and social care professionals in Finland) and the National Professional Association for the interests of experts and managers in health care (TAJA) to their members under 65 years of age, including 58,276 nurses, midwives, and public health nurses representing 72% of the eligible population [29]. One reminder was sent to those who did not respond. A more detailed description of the study can be found elsewhere [29]. Altogether, 10,094 registered nurses opened the link and 3912 responded. Of those who responded, 302 answered that they did not perceive themselves as fit to answer the questionnaire because they had not worked as registered nurses for a long time. Thus, the final sample included 3610 respondents (93.1% women) aged between 22 and 65 years (mean 45.7, SD 11.0) [29]. The sample was representative of the eligible population in terms of the regionality and employment sector. Women were slightly overrepresented, and those under 40 years of age were slightly underrepresented [29]. According to a power analysis conducted using WebPower, an internet-based tool [30], the study had more than 95% power to detect small effects (f=0.1) with an α level of .05 in a 2×4 analysis of variance (ANOVA). Ethical approval for the study was provided by The Finnish Institute for Health and Welfare (THL/482/6.02.01/2020).

### Measures

The questionnaire items used in the present study can be found in Multimedia Appendix 1.

#### Dependent Variables

*SRIS* was measured with the mean of 2 items, framed in 1 question that asked how often (during the last 6 months) the respondent had been distracted by, worried about, or stressed about (1) constantly changing HIS and (2) difficult, poorly performing information technology (IT) equipment or software. The answers were rated on a 6-point scale ranging from 1 (never) to 6 (constantly). The scale’s reliability (Cronbach α=.74) was established in the present sample. This measure was developed in Finland when examining the health and well-being of physicians. [19-21]. It has previously been associated with, for example, experience in using information systems, cognitive workload, distress, and EHR usability [19,22].

Time pressure was measured with the mean of 2 items (α=.94) measuring how often (during the prior half-year period) a person had been distracted by, worried about, or stressed about (1) constantly being in a hurry and time pressure coming from unfinished work tasks and (2) having too little time to do work properly. The items were rated on a 6-point scale ranging from 1 (never) to 6 (constantly). This measure has been widely used previously and associated, for example, with the nurses’ perceptions on the poor usability of EHRs [5].

Cognitive failures can be defined as “cognitively based errors that occur during the performance of a task that a person is normally successful in executing” [31]. They were measured with 3 items (α=.6) derived from the Workplace Cognitive Failure Scale (WCFS) [32,33]. Our survey included 1 item from each of the 3 dimensions of the WCFS: failure of memory, failure of attention, and failure of action. The chosen items have previously shown the highest loadings for their dimensions [33]. Participants were asked to rate how often they have faced situations at work where they (1) have not been able to remember work-related passwords, sets of numbers, etc. (memory failure); (2) have not fully listened to the instructions or requests they have received (attention failure); and (3) have accidentally started or closed the wrong device, system, or program (action failure). Items were rated on a 5-point scale ranging from 1 (never) to 5 (several times a day).

#### Independent Variables

EHR implementation was measured with a question asking whether the respondent’s work unit had implemented or will implement a new EHR. The response options were (1) yes, within the last 6 months, (2) yes, within last 12 months, (3) no, but within the forthcoming 12 months, and (4) no past or forthcoming implementations within 12 months.

Training was assessed with a question asking whether the respondent had received sufficient training related to the required changes in work practices (such as new electronic documentation and care practices) due to HIS implementations. The answer options ranged between 1 (completely disagree) and 5 (completely agree). This question also included the answer option “cannot answer,” which was coded as missing. The responses were coded as 0=insufficient training (answer options 1-3) and 1=sufficient training (answer options 4-5).

As control variables, gender, age, and employment sectors were also included in the survey. Employment sectors were coded as 1=hospital, 2=primary care, 3=social services, and 4=other.

### Statistical Analysis

The associations of the implementation phase and training with the dependent variables were analyzed with analyses of covariance (in separate analyses for each dependent variable). The analyses were adjusted for age, gender, and employment sector. The interactions of the employment sector with the implementation phase and training for the dependent variables were examined with analyses of covariance adjusted for age, gender, and primary effects (in separate analyses for each interaction and dependent variable). Respondents who had missing data for a given variable were excluded from the analyses of that variable. Thus, due to missing information in some variables, n varied between 3525 and 3610.

To further examine the validity of our dependent variables, we conducted principal components analysis (PCA) through direct oblimin rotation with the items of the dependent variables (SRIS, time pressure, and cognitive failures). Moreover, the analyses of covariance were repeated for analyzing sensitivity using the principal component scores resulting from these analyses as the dependent variables.

## Results

### Demographics

The characteristics of the study population are given in Table 1. Approximately 25% (834/3610) of the respondents had experienced EHR implementation within the preceding 6 months, 13% (476/3610) within the preceding 12 months, and 20% (714/3610) reported forthcoming EHR implementation within 12 months. More than half of the respondents (1894/3573) reported insufficient training regarding changes required in the way of working due to HIS implementations.

**Table 1:** Basic background characteristics of the study sample (N=3610^a^).

Characteristic		Value
**Gender, n (%)**		
	Women	3340 (93.1)
	Men	249 (6.9)
**Employment sector, n (%)**		
	Hospital	1903 (52.7)
	Primary care	795 (22)
	Social services	445 (12.3)
	Other	467 (12.9)
**EHR^b^ implementation phase, n (%)**		
	Yes, within the last 6 months	834 (23.1)
	Yes, within the last 12 months	476 (13.2)
	No, but forthcoming within the next 12 months	714 (19.8)
	No	1586 (43.9)
**Training related to implementation, n (%)**		
	Insufficient	1894 (53)
	Sufficient	1679 (47)
Age,^c^ mean (SD)		45.68 (10.97)
Stress related to information systems,^c^ mean (SD)		3.7 (1.13)
Time pressure,^d^ mean (SD)		4.54 (1.12)
Cognitive failures,^e^ mean (SD)		1.88 (0.5)

^a^Due to missing information in some variables, n varies between 3573 and 3610.

^b^EHR: electronic health record.

^c^Ranged between 22 and 67.

^d^Ranged between 1 and 6.

^e^Ranged between 1 and 5.

### Main Effects for SRIS

Table 2 shows the results of analyses of covariance. Age, gender, employment sector, implementation phase, and training were all associated with SRIS. Women had higher levels of SRIS than men. Higher age was associated with higher levels of SRIS. The highest level of SRIS was in hospitals and the lowest was in social care. As observed in Table 3, those who had experienced EHR implementation within the preceding 6 months perceived the highest levels of SRIS, whereas those who did not have to experience forthcoming or prior implementations within 12 months perceived the lowest levels of SRIS. Those who perceived that they had received sufficient training had lower levels of SRIS compared to those who perceived that they had not received sufficient training.

**Table 2:** Associations among explanatory factors with stress related to information systems, time pressure, and cognitive failures (analysis of covariance^a^).

Variable	SRIS^b^		Time pressure		Cognitive failures	
	*F* test *(df)*	*P* value	*F* test *(df)*	*P* value	*F* test *(df)*	*P* value
Age	15.47 (1)	<.001	30.31 (1)	<.001	4.96 (1)	.03
Gender	9.50 (1)	.003	20.57 (1)	<.001	0.21 (1)	.65
Sector	24.14 (3)	<.001	1.77 (3)	.23	1.36 (3)	.25
Implementation	118.43 (3)	<.001	3.05 (3)	.03	5.36 (3)	.001
Training	153.40 (1)	<.001	80.95 (1)	<.001	34.96 (1)	<.001
R^2^	0.165	N/A^c^	0.033	N/A	0.015	N/A

^a^Due to missing information in some variables, n varies between 3525 and 3546.

^b^SRIS: stress related to information systems.

^c^N/A: not applicable.

**Table 3:** Estimated marginal means^a^ of stress related to information systems, time pressure, and cognitive failures according to implementation phase.^b^

Implementation phase	SRIS^c^			Time pressure			Cognitive failures	
	Mean	SE	Mean		SE	Mean		SE
Within the last 6 months	4.07	0.05	4.55		0.06	1.87		0.03
Within the last 12 months	3.76	0.06	4.45		0.06	1.94		0.03
Forthcoming within the next 12 months	3.44	0.05	4.45		0.06	1.86		0.03
No implementations	3.26	0.04	4.41		0.05	1.84		0.02

^a^Adjusted for age, gender, employment sector, and training.

^b^Due to missing information in some variables, n varies between 3525 and 3546.

^c^SRIS: stress related to information systems.

### Main Effects for Time Pressure

Age, gender, implementation phase, and training were associated with time pressure. Women had higher levels of time pressure than men. Higher age was associated with lower levels of time pressure. Those who had experienced EHR implementations within the preceding 6 months had the highest levels of time pressure, whereas those who did not have to experience forthcoming implementations or postimplementation outcomes within 12 months had the lowest levels of time pressure, as indicated in Table 3. Sufficient training was associated with low levels of time pressure.

### Main Effects for Cognitive Failures

Age, implementation phase, and training were associated with cognitive failures. Higher age was associated with lower levels of cognitive failures. Those who had experienced EHR implementation within the preceding 12 months had higher levels of cognitive failures compared to other groups (Table 3). Those who perceived that they had undergone sufficient training had lower levels of cognitive failures compared to those who perceived that they had not had sufficient training.

### Interactions With the Employment Sector

**Figure 1:**
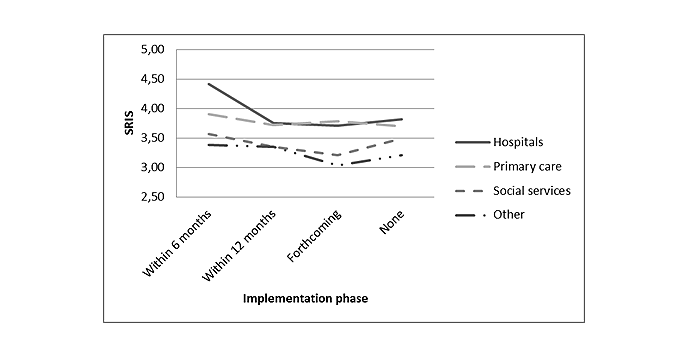
Interaction between implementation phase and employment sectors for stress related to information systems (*P*<.001). SRIS: stress related to information systems.

**Figure 2:**
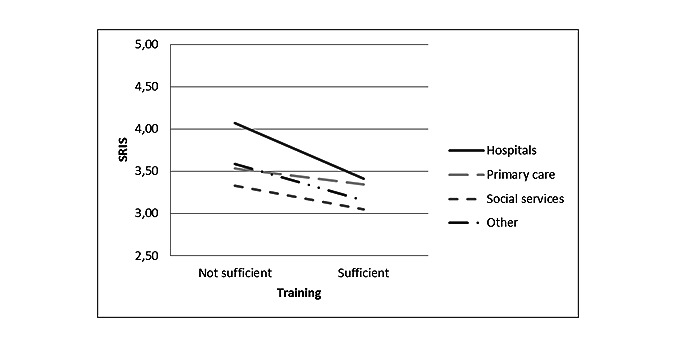
Interaction between training and employment sectors for stress related to information systems (*P*<.001). SRIS: stress related to information systems.

The interaction between the implementation phase and employment sector was significant for SRIS (*F*_9_=4.32, *P*<.001). As observed in Figure 1, implementation in hospitals within 6 months is associated with higher SRIS levels than other sectors (n of the different groups varied between 49 and 715). Moreover, the interaction between training and the employment sector was significant for SRIS (*F*_3_=10.18, *P*<.001). SRIS levels in hospitals were particularly high if insufficient training was perceived, as shown in Figure 2.

**Figure 3:**
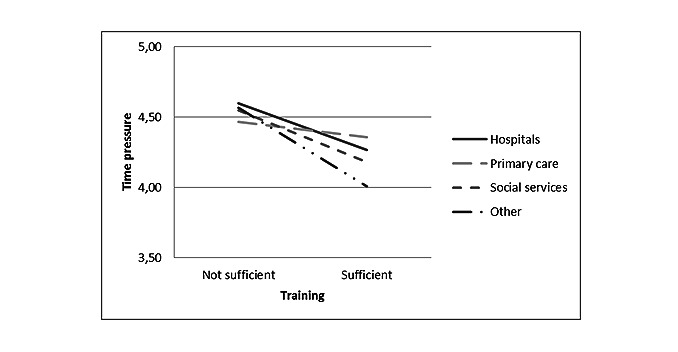
Interaction between training and employment sectors for time pressure (*P*=.006).

The interaction between training and the employment sector was significant for time pressure (*F*_3_=4.18, *P*=.006). In primary care, sufficient training was not so strongly associated with time pressure, whereas in all other sectors, time pressure levels were low if training was perceived to be sufficient, as observed in Figure 3. The interaction between the implementation phase and employment sector was not significant for time pressure.

The interactions of the employment sector with the implementation phase and training were not significant for cognitive failures.

### Sensitivity Analyses

The results of PCA are given in Multimedia Appendix 2. Results showed that the principal components resulting from the analysis were similar, as the original measures used (SRIS, time pressure, and cognitive failures) and the items loaded well with these principal components. ANOVA conducted with the principal component scores resulting from PCA as the dependent variables showed results corresponding with the analyses conducted using original variables (see Multimedia Appendix 3). Moreover, the interaction results with the principal components scores were congruent with the results from the original analyses.

## Discussion

### Principal Results

Our results show that EHR implementations have the potential to decrease the well-being of registered nurses. More specifically, we found that the highest levels of stress related to poorly functioning information systems and time pressure were experienced among those who had experienced EHR implementation within the preceding 6 months. In particular, recent implementations were strongly associated with high levels of SRIS in hospitals. The highest levels of cognitive failures were instead experienced among those who had experienced EHR implementation within the preceding 6 to 12 months. The lowest levels of SRIS, time pressure, and cognitive failures were experienced among those who did not have any past or forthcoming implementations within 12 months.

Sufficient training related to implementations appears extremely crucial for nurses and is associated with improved well-being. Those nurses who perceived that they had received sufficient training related to the changes required in work practices due to HIS implementations experienced less SRIS, time pressure, and cognitive failures. The highest levels of SRIS were among those nurses who worked in hospitals and did not receive sufficient training.

### Limitations

We used self-reported measures, which always poses a question related to problems associated with an inflation of the strengths of relationships and common method variance. The reliabilities of our scales were good, except that the reliability of the cognitive failures scale was 0.6, which can be considered low. However, this reliability can still be considered acceptable because the scale included only 3 items [34]. Moreover, we used cross-sectional survey data; thus, causal inferences cannot be drawn from our results. It is possible that nurses who are more competent in using EHRs also learn to use the new system more quickly and are also more likely to perceive that they have received sufficient training.

In addition, a major limitation of this study is the low explanatory strength of the independent variables used for time pressure and the cognitive failures variables, which can be seen in the low R² values of the analyses of covariance regarding these variables. Consequently, our results should be interpreted with caution and future studies with high-quality validated measures are still needed on this subject. However, even the small effect size may be noteworthy, and it has been suggested that even though the size of the effect in psychological research would be very small, it may potentially be very consequential in the long run [35]. The effect size was larger when explaining the SRIS variable, which is logical given that when a new EHR is implemented, the strain and stress observed among nurses are not surprising.

Moreover, although we adjusted our analyses for age, gender, and employment sectors, the possibility of residual confounding cannot be totally eliminated. For example, it is possible that some unknown third variable may have an effect on the stress and perceived training level and consequently explain the relationship between training and our dependent variables. Finland is a country with universal health care for all residents and one of the forerunners in the digitalization of health care [36]. Therefore, we must be cautious in generalizing our findings to countries with dissimilar health care systems or HIS.

We used a rather large sample of registered nurses (3610 nurses), which was obtained from the registers of academic associations and trade unions and may have affected the representativeness of the sample. The email invitation to participate in the survey was sent to 72% of the eligible population and our sample represented the eligible population in terms of the regionality and employment sector but included a slightly disproportionate cohort of women and those over 40 years of age [29]. The data were collected in the spring of 2020 (March to April) at the time when the COVID-19 pandemic gained prominence in Finland. The most stringent restrictions so far were implemented in the middle of March 2020. Therefore, only 1 reminder was sent to those who had not responded to the invite. These circumstances may have had an effect on the results, especially in those hospitals that were most strongly affected by the pandemic.

### Comparison With Prior Work

Our results show that EHR implementations may endanger the well-being of nurses. This is congruent with previous findings showing that EHR implementation is associated with decreased interdisciplinary communication, a high demand on work time, and low perceived quality of care among nurses [4]. Correspondingly, nurses have been found to experience stress due to added work, along with concerns about security and encountering poor cooperation in the early stage after the implementation of the nursing information system [15]. It has also been shown previously that implementation seems to induce stress, frustration, and feelings of incompetency, especially among those nurses who have problems with tasks requiring digital skills [37].

According to our results, recent implementations occurring especially within the preceding 6 months seem to induce SRIS and time pressure. Previous studies have also obtained congruent findings [6,12,13]. The dissatisfaction seems to be the greatest soon after implementation and then declines, moving toward greater acceptance 12 months after implementation [12,13]. However, we found that cognitive failures were the highest from 6 to 12 months after implementation. A previous study has found that cognitive workload among nurses is the highest just after the EHR implementation and then returns toward baseline after 4 months [6]. There are many possible reasons for our finding that cognitive failures were the highest 6 to 12 months after implementation. For example, it may pertain to implementation-related support from vendors and organizations. It is also possible that nurses are protected from other cognitively burdensome tasks immediately after implementation.

Our findings show that besides implementation aspects related to the change from paper-based documentation to EHRs, implementation factors regarding the change from one EHR to another EHR may affect employees’ well-being. Traditionally, the focus of previous studies has been on the effects of transitioning from paper-based documentation to EHRs [4,6,12]; however, studies focusing on the effects of transitioning from one EHR to another are emerging [13]. In future, we may expect research findings focusing on the transition from one EHR to another, given the widespread use of EHRs in developed countries.

Our results suggest that organizations should implement measures to decrease the negative impact of implementations. A meta-analysis suggested a re-engineering approach to better integrate HIS implementation in health care workflows [3]. During re-engineering, organizations should examine and consider restructuring their work processes related to operational factors and infrastructure in a manner that could enable them to optimally use HIS functions. Moreover, improving the usability of systems would support the implementation, decrease the need for training, and improve employee well-being [22,38,39]. The users should be allowed a transition period, giving them time to understand and appreciate the outcome of the system implementation [3]. Further, user involvement, strong leadership, project management techniques, and standards are important in ensuring successful implementation [3,39,40]. To improve the experience of nurses in the beginning stage, it would be important to commit the nurses to the system design early on [15].

Our findings highlight the importance of proper training related to implementations. According to our findings, it may be possible to decrease the negative ramifications of implementations on nurses’ well-being and cognitive functioning with sufficient training. However, training is insufficient in many cases. In our study, 53% reported that training was insufficient. In another study, 62% of health care staff reported that they had not received enough training related to inpatient portal implementation [16]. Moreover, nurses have indicated in focus groups that they had insufficient training related to the nursing information systems [15].

Our results showed some variations according to the employment sectors. In hospitals, the stress levels associated with implementations were the highest and the training related to systems was especially important. This finding effectively reflects the previous findings among physicians showing that attitudes toward EHRs are most critical in hospitals [41-43]. It is also possible that hospitals especially experience insufficiencies in IT support, given that nurses in hospitals often also work outside of office hours. A previous study has suggested that in addition to training, organizations should also identify and appoint champions who could learn more thoroughly and teach others how to use different systems [44].

In primary care, it seems that sufficient training is also inadequate to buffer against time pressure. In Finnish primary care, one of the reasons for time pressure among nurses is that primary care involves accessibility problems and long waiting times [45]. A previous study among Finnish nurses showed that technical problems and poor user-friendliness of the EHRs are associated with high time pressure [5]. Thus, in primary care, tackling the problems associated with the usability of the systems that are implemented might be important in terms of time pressure.

Our results show that sufficient training related to implementations is highly important. It might be beneficial to offer training and other support during the whole implementation period, that is, before, during, and after the implementation [46,47]. The amount of education has been found to be positively correlated with nurses ’attitudes and behaviors toward the implemented IS; thus, it is important that organizations provide quickly and easily accessible in-house support and proactive training in the use of HIS [48]. In addition, evidence indicates that it is important to consider which training methods would best support professionals in developing the necessary skills and using the systems. Adequate education and training encourage employees to use HIS, which are prerequisites for benefiting from implementations [3]. According to our results, training related to changing work practices due to HIS implementations is particularly important. Additionally, training featuring improvements such as keyboard entry skills, redesigning workflow, and improving interdisciplinary communication are considered necessary [15].

We suggest that training should be planned carefully in advance, including basic training at least 2 to 3 weeks before implementation. After implementation, training should continue for several weeks, following which the authorities must assess whether more training is needed. Moreover, it is important to provide time for nurses to learn to use the systems in practice.

### Conclusions

The present study shows that EHR-to-EHR implementations and insufficient training related to the implementations may impair nurses’ well-being and even lead to cognitive failures. Thus, it is crucial that organizations implement measures to decrease the negative ramifications of implementations on their employees. This would be very important in all sectors, but especially in hospitals.

## Appendix

Multimedia Appendix 1 Measures used in the study.

Multimedia Appendix 2 Results of principal component analysis with direct oblimin rotation: component loadings and variance explained.

Multimedia Appendix 3 Association of explanatory factors with principal component scores for stress related to information systems, time pressure, and cognitive failures (analysis of covariance).

## Abbreviations

EHRs: electronic health records
HIS: health information systems
IT: information technology
PCA: principal components analysis
SRIS: stress related to information systems
WCFS: Workplace Cognitive Failure Scale
