# Supplementary material for: Electronic Health Record Implementations and Insufficient Training Endanger Nurses’ Well-being: Cross-sectional Survey Study
Source: J Med Internet Res. 2021 Dec 23;23(12):e27096. doi: 10.2196/27096 (PMC8738988; doi:10.2196/27096)
Supplement: Multimedia Appendix 1 [file jmir_v23i12e27096_app1.docx]

**Multimedia Appendix 1**

**Measures used in the study**

**Dependent variables**

***Stress related to information systems (SRIS):***

*How often has each of the issues mentioned below clearly disturbed, worried or burdened you at work during the past 6 months?*

1. Constantly changing information systems
2. Difficult, poorly performing IT equipment/software

Response options:

1. Never
2. Very rarely
3. Quite rarely
4. Quite often
5. Very often
6. Constantly

***Time pressure***

*How often has each of the issues mentioned below clearly disturbed, worried or burdened you at work during the past 6 months?*

1. Being in a constant hurry and time pressure coming from unfinished work tasks
2. Having too little time to do work properly

Response options:

1. Never
2. Very rarely
3. Quite rarely
4. Quite often
5. Very often
6. Constantly

***Cognitive failures:***

*Think about your work and estimate how often you have faced situations at work where you:*

1. Have not remembered work-related password, set of numbers etc .
2. Have not fully listened to the instructions or requests I have received.
3. Have accidentally started or closed the wrong device, system or program.

Response options:

1. Never
2. Monthly or less
3. Weekly
4. Daily
5. Several times a day

**Independent variables**

***EHR Implementation:***

*Has your unit implemented or will implement in the future new electronic health record*

Response options:

1. Yes, within past 6 months
2. Yes, within past 12 months
3. No, but forthcoming within next 12 months
4. No past or forthcoming implementations within 12 months

***Training:***

*I have received sufficiently training related to required change in work practices (such as new electronic documentation and care practices) due to information systems implementations.*

Response options:

1. Completely disagree
2. Somewhat disagree
3. Don’t agree or disagree
4. Somewhat agree
5. Completely agree
6. Cannot answer (coded as missing)

**Control variables**

***Employment sector:***

*Principal occupations’ employment sector*

Coded as:

1. Hospitals
2. Primary care
3. Social care
4. Other (e.g., private, foundation etc.)
