# Supplementary material for: Electronic Health Record Implementations and Insufficient Training Endanger Nurses’ Well-being: Cross-sectional Survey Study
Source: J Med Internet Res. 2021 Dec 23;23(12):e27096. doi: 10.2196/27096 (PMC8738988; doi:10.2196/27096)
Supplement: Multimedia Appendix 2 [file jmir_v23i12e27096_app2.docx]

| Multimedia Appendix 2. Results of principal components analysis with direct oblimin rotation: component loadings and variance explained | | | |
| --- | --- | --- | --- |
| Item | PC 1  (time pressure) | PC 2  (cognitive failures) | PC 3  (SRIS) |
| TP: constant hurry | .97 |  |  |
| TP: too little time | .96 |  |  |
| CF: not fully listened instructions |  | .78 |  |
| CF: accidentally started/closed wrong device |  | .75 |  |
| CF: not remembered work-related password etc. |  | .71 |  |
| SRIS: constantly changing IS |  |  | -.90 |
| SRIS: difficult, poorly performing IT |  |  | -.87 |
| Explained variance, % (Total 73.45%) | 38.46 | 20.77 | 14.23 |
| PC= principal component, TP = time pressure, CF = cognitive failures, SRIS = stress related to information systems, IS = information systems, IT = information technology | | | |
