# Supplementary material for: Electronic Health Record Implementations and Insufficient Training Endanger Nurses’ Well-being: Cross-sectional Survey Study
Source: J Med Internet Res. 2021 Dec 23;23(12):e27096. doi: 10.2196/27096 (PMC8738988; doi:10.2196/27096)
Supplement: Multimedia Appendix 3 [file jmir_v23i12e27096_app3.docx]

| Multimedia Appendix 3. The association of explanatory factors with principal component scores for SRIS, time pressure, and cognitive failures (analysis of covariance) | | | | | | |  |
| --- | --- | --- | --- | --- | --- | --- | --- |
| Variable | SRIS | | Time pressure | | Cognitive failures | |  |
|  | *F(df)* | *P* | *F (df)* | *P* | *F(df)* | *P* |  |
| Age | 10.80(1) | .001 | 34.40(1) | <.001 | 3.98(1) | .046 |  |
| Gender | 9.73(1) | .002 | 16.76(1) | <.001 | 0.12(1) | .730 |  |
| Sector | 20.67(3) | <.001 | 1.63(3) | .181 | 1.09(3) | .351 |  |
| Implementation | 107.96(3) | <.001 | 2.77(3) | .040 | 5.27(3) | .001 |  |
| Training | 143.70(1) | <.001 | 62.99(1) | <.001 | 33.03(1) | <.001 |  |
| R^2^ | .162 |  | .034 |  | .014 |  |  |
| SRIS=stress related to information systems | | | | | | |  |
